# Sedentary behaviour in patients with rheumatoid arthritis: A qualitative study

**DOI:** 10.3402/qhw.v10.28578

**Published:** 2015-10-12

**Authors:** Tanja Thomsen, Nina Beyer, Mette Aadahl, Merete L. Hetland, Katrine Løppenthin, Julie Midtgaard, Bente A. Esbensen

**Affiliations:** 1 Copenhagen Centre for Arthritis Research, Centre for Rheumatology and Spine Diseases VRR, Rigshospitalet, University of Copenhagen, Copenhagen, Denmark; 2 Musculoskeletal Rehabilitation Research Unit, Bispebjerg and Frederiksberg Hospitals, University of Copenhagen, Copenhagen, Denmark; 3 Department of Clinical Medicine, Faculty of Health and Medical Sciences, University of Copenhagen, Copenhagen, Denmark; 4 Research Centre for Prevention and Health, Glostrup Hospital, University of Copenhagen, Copenhagen, Denmark; 5 Section of Social Medicine, Department of Public Health, University of Copenhagen, Copenhagen, Denmark; 6 University Hospitals Centre for Health Research, Rigshospitalet, University of Copenhagen, Copenhagen, Denmark

**Keywords:** Sitting time, fatigue, pain, everyday life, chronic disease, adaptation, lifestyle

## Abstract

**Background:**

Despite increasing interest in investigating sedentary behaviour (SB) in the general population and in patients with rheumatoid arthritis (RA), there is little documentation of the subjective experiences of SB in patients with RA. This study aimed to examine how patients with RA describe their daily SB.

**Methods:**

Fifteen patients with RA (10 women and 5 men) from 23 to 73 years of age and with a disease duration ranging from 4 to 27 years were interviewed following a semi-structured interview guide. Data were analysed using the content analysis method described by Graneheim.

**Results:**

SB appeared in three categories covering: 1) *A constant battle between good and bad days*; SB could be a consequence of RA in terms of days with pronounced pain and fatigue resulting in many hours of SB. 2) *Adaptation to everyday life*; living with the unpredictability of RA included constant modification of physical activity level causing increase in SB, especially during periods of disease flare. Prioritizing and planning of SB also functioned as part of self-management strategies. 3) *It has nothing to do with my arthritis*; for some patients, SB was not related to RA, but simply reflected a way of living independent of the disease.

**Conclusions:**

SB is perceived, motivated, and performed differently in patients with RA. An individually tailored approach may be essential in understanding and encouraging patients’ motivation towards sustainable change in SB and activity patterns.

Sedentary behaviour (SB), defined as any waking behaviour characterized by energy expenditure <1.5 METs (metabolic equivalents) while in a sitting or reclining posture, has become increasingly prevalent in industrialized countries (Grontved & Hu, 2011; Sedentary Behaviour Research Network, 2012; Thorp, Owen, Neuhaus, & Dunstan, 2011). Objective measurements of physical activity and SB in patients with rheumatoid arthritis (RA) show that these patients spend a high proportion of the day in SB (Paul et al., 2014; Prioreschi, Hodkinson, Avidon, Tikly, & McVeigh, 2013). Prospective studies have found SB to be associated with increased risk of cardiovascular diseases, obesity, type 2 diabetes, and all-cause mortality in the general population (Dunstan et al., 2010; Grontved & Hu, 2011; Hamilton, Hamilton, & Zderic, 2007; Manns, Dunstan, Owen, & Healy, 2012; Owen, Healy, Matthews, & Dunstan, 2010). Patients with RA already have an increased risk of cardiovascular disease and premature death caused by the chronic inflammatory rheumatic disease itself (Kerola et al., 2013). The most frequently used measure of SB is television viewing and thus most documentation of the prevalence of SB in relation to health is based on SB during leisure time; however, SB also occurs in other domains, for example, while sitting at work or during transport (Owen et al., 2011). Despite a well-established association between physical activity and health benefits in both the general population and in patients with RA, the negative health effects of SB have been found even in individuals who meet recommended levels of daily moderate to vigorous physical activity (MVPA) (Healy et al., 2008), which covers a variety of activities from brisk walking and gardening to competitive sports. Feasibility studies and randomized controlled clinical trials have investigated the effect of reducing SB in sedentary office workers (Chau et al., 2014; Neuhaus, Healy, Dunstan, Owen, & Eakin, 2014), and in older (Gardiner, Eakin, Healy, & Owen, 2011), overweight and obese (Carr, Karvinen, Peavler, Smith, & Cangelosi, 2013; Kozey-Keadle, Libertine, Staudenmayer, & Freedson, 2012; Otten, Jones, Littenberg, & Harvey-Berino, 2009) adults and have shown positive results including reduction in daily sitting time and workplace sitting time, increased light intensity physical activity, and increased energy expenditure (Carr et al., 2013; Chau et al., 2014; Gardiner et al., 2011; Kozey-Keadle et al., 2012; Neuhaus et al., 2014; Otten et al., 2009). Cross-sectional studies have investigated SB in patients with chronic diseases and mobility disabilities, including those with strokes, Parkinson's disease, multiple sclerosis, and RA, and they generally found that these patients spent a high proportion of the day sitting (Chastin et al., 2010; Ellis & Motl, 2013; Moore et al., 2013; Prioreschi et al., 2013). In patients with RA, the amount of objectively measured total daily sitting and lying time has been found to be statistically significantly higher compared with healthy controls (18 h and 50 min vs. 17 h and 43 min) (19 participants in each group) (Paul et al., 2014). In a Danish cohort of patients with RA (*N*=438), 27% reported being primarily sedentary (Loppenthin et al., 2015). In addition, 67% of Danish patients with RA do not comply with public health recommendations for daily MVPA (Sokka et al., 2008). Reported worries about causing harm to the joints, painful joints, and severe fatigue are some of the reasons for not meeting physical activity recommendations in patients with RA (Wikstrom, Book, & Jacobsson, 2006; Wilcox et al., 2006). Aiming to reduce SB, rather than solely promoting MVPA, may be a health promotion strategy that is feasible and suitable in patients with RA. However, there is a lack of literature documenting how SB is experienced and performed in patients with RA. In order to target an intervention with the purpose of reducing SB in patients with RA, this study aimed to examine how patients with RA describe their daily SB.

## Methods

### Design

A qualitative, explorative, and descriptive design drawing on hermeneutical reflection (Malterud, 2008) was chosen as most suitable to capture the unique perspectives of how daily SB was experienced by patients with RA and thereby to gain understanding of their situation. Understanding other human beings involves recording their experiences (Malterud, 2008); hence, interviewing was chosen as the method for data collection.

### Sampling and recruitment

Patients who had participated in a cross-sectional questionnaire study of physical activity (*N*=438) (Loppenthin et al., 2015), were recruited from the outpatient rheumatology clinic of Glostrup Hospital. Initially, the following selection criteria were applied: 1) meeting the 1987 American College of Rheumatology/European League Against Rheumatism criteria for RA, and 2) self-reported leisure time SB >4 h per day assessed by the Physical Activity Scale (PAS 2.1) (Aadahl & Jorgensen, 2003). This left 140 eligible patients. From those, we selected patients to cover all levels of self-reported daily leisure time SB, ranging from 4 to 10 h. Based on these criteria, we sent letters with information about the study to 18 patients. After a few days the first author (TT) contacted them by telephone and asked if they were interested in participating. Sixteen patients agreed to participate; two chose not to do so due to lack of time. One of the patients later withdrew, due to critical illness of a family member, before the interview had been conducted, leaving 15 patients to be interviewed.

### Interviews

Semi-structured interviews were conducted by TT from May to August 2012. Eight patients chose to be interviewed in their homes, five in TT's office, and two preferred to be interviewed at the rheumatology outpatient clinic. Each patient was encouraged to describe a typical day with the opening question: “Please include all activities in describing an average weekday from getting out of bed in the morning until bedtime.” BAE and TT developed a semi-structured interview guide, which acted as a trigger and an inspiration for further conversation. The interview guide was piloted in interview 1 and only minor changes were done subsequently. The guide is presented in Table I. Each interview ended with the patient answering questions about sociodemographic status, duration of RA, functional independence, and medical treatment (Table II).

**Table I:** Interview guide

Opening question	Please, describe an average weekday for you, from you get out of bed in the morning until bedtime. Please, include all your activities
Focus	
Physical activity/exercise	Before and after diagnosed with RA How much, what, when Experiences, thoughts, reasoning
Daily sitting time	Before and after diagnosed with RA When, what, where, why, with whom? Experiences, thoughts, reasoning
Factors/persons influencing daily sitting time	In everyday life Who, what, when, why? Experiences, thoughts, reasoning

**Table II:** Characteristics of the participants (*N*=15)

ID #	Sex	Age	RA-duration (years)	Treatment^a^	Independency	Leisure time sitting hours/day	Cohabiting	Residence	Employment	Education
1	F	48	8	BT	Independent No difficulty	8	With partner	Apartment	No job	Secondary
2	M	71	13	BT	Independent No difficulty	6	With partner	House	Retirement—age	Minimal
3	F	61	12	DMARDs	Independent No difficulty	5	With partner	House	Retirement—age	Secondary
4	F	61	16	DMARDs	Independent No difficulty	7	With partner	House	Retirement—disease	Minimal
5	M	73	27	DMARDs	Independent with difficulty	8	With partner	House	Retirement—age	No
6	F	66	10	DMARDs	Independent No difficulty	6	Single	Apartment	Retirement—age	Minimal
7	M	68	9	BT	Independent No difficulty	7	With partner	House	Retirement—age	University
8	F	66	17	DMARDs	Independent No difficulty	7	Widow	Apartment	Retirement—disease	Secondary
9	F	37	6	BT	Independent No difficulty	5	With partner	Apartment	Full time	University
10	F	67	21	DMARDs	Independent with difficulty	10	Single	Apartment	Retirement—age	Secondary
11	M	23	4	BT	Independent No difficulty	5	With partner and child	House	Part time	Secondary
12	F	65	22	Glucocorticoids	Independent with difficulty	10	Widow	Apartment	Retirement—disease	No
13	F	45	8	BT	Independent No difficulty	9	With partner and children	Apartment	Part time	Secondary
14	F	46	11	BT	Independent No difficulty	6	Single with child	Apartment	Full time	University
15	M	56	14	BT	Independent No difficulty	5	With partner and child	House	Full time	Secondary

a DMARD=disease-modifying anti-rheumatic drug; BT=biological treatment (TNF-a).

The interviews lasted between 30 and 90 min and were digitally recorded.

### Analysis

Each interview was transcribed verbatim and Nvivo software (version 9.0) was used to facilitate systematic structuring of the data. Interview data were analysed separately by two researchers (BAE and TT) applying the method of qualitative content analysis as described by Graneheim (Graneheim & Lundman, 2004). The analysis was a four-step descriptive process. First, we read the text several times in order to get an overall impression of data. Second, we identified aim-related content and divided it into content units (Graneheim & Lundman, 2004). Third, frequently occurring content units were condensed into content areas and labelled with a code representing different experiences in relation to SB. We defined subcategories to enable the content units to fit together in the most meaningful way. In addition, the differences between the subcategories had to be clear and the authors made efforts to keep subcategories mutually exclusive (Graneheim & Lundman, 2004). The subcategories were discussed between the two researchers who reached agreement on abstracting them into three main categories. These were based on recurring regularity within the subcategories or cutting across them. Each of the main categories may be considered as the latent content of the text (Graneheim & Lundman, 2004). Finally, the other authors acted as peer reviewers in discussing and commenting the findings reached by BAE and TT.

### Ethics

All study participants provided informed consent. The study was reported to the Danish Data Protection Agency (ref. nb. 711-1-08) and The Health Research Ethics Committee System in Denmark (ref. nb. H-4-2014-FSP) and was performed in compliance with the Helsinki Declaration.

## Results

Ten women and five men participated in the study; their characteristics are presented in Table II. The patients were between 23 and 73 years of age and their disease duration ranged between 4 and 27 years. Twelve of the patients were functionally independent whereas three were functionally independent but with some disability regarding activities of daily living. The leisure time SB ranged between 5 and 10 h per day.

Based on the analysis, three categories and mutually related subcategories were identified; 1) *A constant battle between good and bad days*, that is, the battle between disease flares and symptoms effectively controlled by the medical treatment, 2) *Adaptation to everyday life*, including means to protect the joints by sitting breaks and rest time, and 3) *It has nothing to do with my arthritis*, that is, SB is influenced by co-morbidities and social relations. Categories and subcategories are shown in Table III.

**Table III:** Categories and subcategories

Category	Subcategory
A constant battle between good and bad days	Being dependent on efficient medical treatment
	When symptoms dominate
Adaptation to everyday life	The body signals a need for sitting time
	Protection of the joints is essential
	Awareness of rest, movement, and sitting time
It has nothing to do with my arthritis	Co-morbidities are influencing sitting time
	Simply a way of living
	Social relations contribute to increased and decreased sitting time

### A constant battle between good and bad days

#### Being dependent on efficient medical treatment

The fluctuation of the disease, varying between good days and bad days, was described as a constant battle in the body. It was a battle between disease symptoms and the effect of the medical treatment on symptoms. Being without medical treatment, receiving ineffective treatment, or being in the last days before a new dose of medicine at the outpatient clinic meant high levels of pain and fatigue and consequently many bad days and more daily sitting time. Conversely, when the patients responded efficiently to the treatment it was experienced as something controlling the body which was referred to as good days.

My new medication revolutionizes my body. I am a whole new person. Right after my medicine dose at the clinic I feel so good. I move more and my mood is better. On bad days, the arthritis still breaks out and takes over my body. It is in all my joints and it hurts all over. Then I sit and do whatever, e.g., my crosswords or reading a book. So actually it is the medicine that controls my life. So it is. (P#2)

Several of the patients described periods of time with insufficient medical treatment, where they had been dependent on help from family members in performing daily activities and where they had spent most of their leisure time sitting. Now, being on an effective treatment regime they experienced only little limitation in daily activities.

#### When symptoms dominate

Despite a general feeling that the RA was well controlled by treatment, all patients described days when the RA was dominating in terms of severe fatigue and joint pain. On these days, daily activities would be extremely exhausting. Activities that included a lot of standing and walking were especially strenuous, for example, a dishwashing was described as causing tears because of aching feet. Patients who were working described how on certain days it would require all their energy merely to go to work.

I am extremely tired. Some evenings when I return from work and sit on the couch and turn on the TV I simply pass out. It is like I use all my strength at work. All energy is gone. My movements cause me pain, so I use more efforts during the day, which you compensate for at night. That's how I see it. (P#15)

As a consequence of fatigue they would give up evening activities such as going out for an evening walk or gardening, which was otherwise part of their day-to-day leisure time activities. Fatigue especially was experienced as a limitation preventing patients from engaging in daily activities. It was described as something that would cause the body to feel heavy, not possible to sleep away, as an infection or as a woman put it.

My body really, really hurts and I am so tired. It feels like I am carrying two buckets of water all the time. My arms just hang. That's how tired I am. It annoys me, because I do not want to be that tired, but you are tired. (P#8)

For all patients, the physical activity level would be minimal on days with a lot of joint pain and fatigue, resulting in plenty of SB such as watching TV, reading, doing cross-words, or needlework.

These days could come without warning, which made it difficult to plan, for example, social activities. Several of the patients described how they had cancelled plans, for example, dinner plans, concerts, family days because of sudden rushes of fatigue forcing them to lie down and cancel the appointment. This also affected many of the patients psychologically in terms of feelings of irritation and frustrations when pain and fatigue prevented them from doing things they were perfectly capable of doing at other times.

Days with high levels of pain and fatigue could also be experienced as particularly isolating because RA either made it difficult to leave home or the patients deliberately chose to be by themselves at home on those days.

I try to protect myself and hide at home, because I am so tired all the time. And I cannot motivate myself to do anything. So actually my home base is my own personal hell some days. (P#1)

### Adaptation to everyday life

#### The body signals a need for sitting time

On some days, RA forced the patients to take more sitting breaks between day-to-day activities, than they used to take prior to onset of the disease. A daily walk to town sometimes had to be interrupted by a sitting break on a bench due to aching feet. Pain and stiffness in the joints were most distinct in the morning, which meant that many of the patients set aside time to sit and let the body come alive. This process could last from 30 min to 2 h and required them either to sit on the couch or, on especially painful mornings, to take analgesics and go back to bed for a while. These ways of handling the mornings were described as a preparation, and were also used on days when the patients were not affected by pain and stiffness; the morning rest had just become part of the daily routine.

#### Protection of the joints is essential

Daily routines were also devised to put as little strain as possible on the joints. For some patients, sitting was considered better for the joints and too much movement would strain the joints and make the patients pay in terms of severe joint pain the following day. This implied a number of daily strategies, for example, always to go by car, never on a bicycle, not to take longer walks than necessary, and never walk upstairs, only downstairs.

My girlfriend and I have put a barstool in the kitchen. That way it is possible for me to sit while cooking. I want to protect myself. By doing all these little things in everyday life I believe it will help me in the long term. Even though the medicine really has improved my everyday life, some days I don't even notice I suffer from RA, I still want to protect myself and not strain my joints. My legs were really aching once. (P#11)

Similar strategies and concerns to protect the joints were described by several patients and they were especially expressed by those patients who had experienced long periods earlier in their RA trajectory with severe symptoms and no effective medical treatment. During those periods, they had been dependent on help from others and had forced themselves to perform daily activities with a minimum of effort and strain on the joints. They were thus used to taking precautions in daily activities in order to function as well as possible and these were maintained even in days where symptoms were absent.

#### Awareness of rest, movement, and sitting time

The awareness of having arthritis was also reflected in the way the patients made use of activity pacing and energy conservation in many domestic activities.

I often sit taking a break while doing household activities, for example changing the bed linen. I take the linen off the bed. Then I sit on my walker for a while before I put the sheet and pillow case on. Then I take another break in the living room, sometimes a whole hour, before I put on the rest of the linen and finally the bed cover. It is in steps. (P#6)

Laundry, cooking, and window cleaning were often done in a similar step-wise way. Sleep was not necessary between activities, but the patients had to sit and do some sedentary activities for a while, for example, reading the newspaper, looking something up on the Internet, or taking a short break in the garden chair before continuing work.

It was essential to prioritize a full day of resting during the week which often meant a day on the couch, for example, watching TV, reading, or doing cross-words. These rest days were often planned beforehand or planned after an exhausting day, such as a very busy work day or one filled with social activities.

I allow myself a weekly day of rest when I have been working a lot. On these days nothing is going on besides TV-watching, eating and maybe reading a book. I do not even shower. Maybe I do a little grocery shopping, but mostly I am just home, doing nothing. (P#14)

These days of resting had become a natural part of life. Many of the patients even felt “punished” with severe fatigue if they had a whole week without ensuring time to rest.

### It has nothing to do with my arthritis

#### Co-morbidities are influencing sitting time

Other diseases present led to sitting time and significantly influenced the patient's everyday life. A few women described osteoporosis as being more limiting for their daily activity level than RA in terms of pain from hips and knees. Cardiovascular diseases and lung diseases were also barriers to daily activities, for example, the weekly grocery shopping or climbing the stairs, and led to increased daily sitting time.

It is not the arthritis that prevents me from mowing our lawn, but my heart does not work properly. Sometimes even the stairs feel overwhelming. Also, the time with the slipped disk was awful. The arthritis was nothing compared to that. I could not do anything. (P#5)

#### Simply a way of living

SB was also described as merely a result of lack of interest in moving too much or because of laziness. Sitting was just considered more comfortable.

I have never been interested in sports. It has always been reading, reading, reading. As soon as there was Windows 3.11 on the computer I plunged into that. Even now, I always sit at the computer. Love my games. I'm lazy. Why go out for a walk if you can sit and play a computer game? Life is about to do what you want to, not to live as long as possible. (P#9)

Overall, the patients also performed a lot of sedentary leisure time tasks, for example, needlework, reading, computer games, and cross-words independent of the presence of symptoms. Neither was the choice of the many sedentary tasks described as due to lack of confidence in one's own ability to reduce daily sitting time, but just as a result of personal interest or circumstances. This also applied to patients who were working or studying. Sitting was just more suitable when working or studying and could include sitting down 8–10 h in front of the computer during long days at work or during preparation for an exam.

#### Social relations contribute to increased and decreased sitting time

Time spent sitting was often shared with family and friends. Morning coffees with friends could turn into a full day of sitting, including doing needlework or other SBs. Patients also described lazy nights on the couch watching movies with their partner. In addition, many TV nights at home could be attributed to a partner's disease.

My wife has a serious lung disease. We are very limited in doing things together. About three times I went to a concert or a movie by myself and threw out one of the tickets. Then I decided that I did not want that any longer, so it had to be DVDs or TV. She cannot do anything anymore. Before, we always went out to concerts. (P#5)

On the other hand, social events or family responsibilities could result in reduced sitting time, for example, when picking up one's child from school and going to the playground or spending a whole day playing with the grandchildren.

## Discussion

The present study is, to our knowledge, the first study to report SB in patients with RA from their perspective. The results illustrate that SB could be a consequence of fluctuations in RA in terms of days with pronounced pain and fatigue. On the other hand, it could also serve as a means of managing everyday life living with RA, and finally daily SB was not always related to RA but simply reflected a way of living.

Daily activity level was found to be dependent on the presence of RA-related symptoms, which is in line with previous findings (Hewlett et al., 2012; Kristiansen, Primdahl, Antoft, & Horslev-Petersen, 2012; Loppenthin et al., 2015). A Danish cross-sectional study found that pain, fatigue, and disease activity in patients with RA were associated with being physically active at a low level (Loppenthin et al., 2015). In our study, pain and fatigue were described as the most dominating symptoms affecting patients physically, mentally, and socially. Prevalence of pain and fatigue, and the unpredictability of the symptoms, prevented the patients from performing their usual activities and from planning ahead, which caused much frustration and increased isolation. This is supported by another qualitative study which also found that social withdrawal was a key feature during RA flares, making participation in normal life unthinkable (Hewlett et al., 2012). However, in our study it was notable that fatigue was considered the greatest limitation to daily activities because of its unpredictable nature and because it implied non-expected needs for rest during a day. By further exploring the patients’ perspectives on fatigue we found that it was experienced differently and was described in metaphors like “an infection” or “carrying two buckets of water.” Use of metaphors in describing RA-related fatigue has also been found in another qualitative study where fatigue was described as “being stuck in the mud” or feeling like a “deflated beach-ball” (Feldthusen, Bjork, Forsblad-d'Elia, & Mannerkorpi, 2013). According to Lakoff and Johnson (1980), use of metaphors is essential in the way we conceptualize our day-to-day experiences. Metaphors help us structure and understand our perceptions, thoughts, and actions in new terms, irreplaceable by other expressions (Lakoff & Johnson, 1980). In turn, metaphors have to be transformed into alternative understandings, and in relation to patients with RA, in both our study and previous research (Feldthusen et al., 2013) we propose that the fatigue metaphors reflected associations towards a heavy burden. This appeared not only to be a physical burden but something which was manifest as a feeling of being held down. In the same way, we believe that the metaphors also gave insights into feelings of lack of freedom at times when fatigue was severe. The metaphors thereby emphasize how intrusive fatigue may be in the everyday life of patients with RA, affecting their physical activity level and everyday life.

Days with fatigue and pain followed by difficulties in performing everyday tasks were described as a recurrent consequence of RA, and as an imbalance in everyday life leading to an increased need for sedentary tasks, sleep, and solitude. This imbalance was partly restored on days when the medical treatment “won” against the RA resulting in only minor limitations in performing everyday tasks and enabling the patients to live as they wanted without symptoms determining the amount of time spent sitting. In a qualitative focus group study including patients with RA, Kristiansen et al. (2012) described that effective medical treatment is perceived as a precondition for maintaining everyday life and functional capacity. In line with Kristiansen et al. (2012), it is hoped that our study provides valuable knowledge to healthcare providers aiming to support patients with RA to make changes in their daily physical activity level. This includes awareness of the unpredictability of fatigue in RA, and the degree to which fatigue and pain can affect physical activity and function at times when the patients are not responding adequately to the medical treatment.

It is interesting to note how consistently the patients described SB as a means to adapt to everyday life. Strategies included putting minimal strain on the joints by doing mostly sedentary tasks, planning sitting time in the morning and in between daily activities, and prioritizing weekly days of rest. These findings fit descriptions given by patients with RA in previous research which explicitly aimed to describe how patients coped with their arthritis in everyday life (Feldthusen et al., 2013; Gronning, Lomundal, Koksvik, & Steinsbekk, 2011; Hewlett et al., 2012; Kristiansen et al., 2012; Ottenvall & Hakansson, 2013). As such, previous qualitative studies (Hewlett et al., 2012; Ottenvall & Hakansson, 2013) have described how patients with RA made practical or mental adjustments on a day-to-day basis resulting in a modified physical activity level in order to meet the consequences of the fluctuating nature of arthritis and to ensure a balance between physical activity and rest. In agreement with two other qualitative studies (Feldthusen et al., 2013; Gronning et al., 2011), these daily adjustments often included avoiding energy-consuming activities, doing the activities at one's own pace, and planning extra time for rest between physical or social activities. In accordance with the study by Gronning et al. (2011), our results illustrate that these adjustments were often initiated as a response to signals from the body such as aching feet or joint stiffness in the morning. Sitting time was built into our patients’ daily routines as a way of managing disease symptoms and conserving energy reflecting the practical, mental, and bodily experiences of living with RA. Based on these experiences, the patients had seemingly developed a strong belief in their own ability to manage their arthritis by taking necessary actions to minimize its negative consequences and to gain balance in everyday life. Actions that could include lots of daily sitting time were not necessarily seen as an unhealthy behaviour, but rather described as daily routines in order to adapt to everyday life.

How essential the daily adjustments had become for the patients’ well-being in everyday life was further illustrated by the finding that the patients were worried about being “punished” by the RA if the planning of rest in between activities was not integrated in their everyday life. This is supported by (Ottenvall & Hakansson, 2013) who found that the conscious planning of keeping a balance between activities and rest had a direct relationship with perception of health in patients with RA. To clarify this point further, it is worth mentioning that the patients in our study described planning and prioritizing SB even on days when they were not affected or limited by symptoms. That reflects the extent to which sitting time had become part of the patients’ daily self-management routines. Based on this finding, healthcare providers should not only recognize these lifelong self-management routines, but also challenge them in supporting changes in activity patterns in patients with RA. It further calls attention to the importance of individualizing future health behaviour interventions in respect for the patient's need for planning and managing everyday life.

The results from our study also call attention to healthcare providers to differentiate between RA-related and RA-independent reasons to be sedentary. Motives for daily sitting time might equally be that the patient's personality, priorities, social roles, spousal relationship, and current life circumstances were more consistent with SB than keeping more physically active. Thus, personal and social factors may influence SB independently of health-related concerns. This has previously been reported in a qualitative study, which included older women, where the main issues related to SB were societal pressure and taking more pleasure in sitting (Chastin, Fitzpatrick, Andrews, & DiCroce, 2014). Identical issues have been applied to barriers to physical activity in patients with diabetes and chronic obstructive pulmonary disease, such as low priority, something more interesting on TV, lack of intrinsic motivation, boredom, and lack of belief in one's ability to perform physical activity (Hartman, Ten Hacken, Boezen, & De Greef, 2013; Thomas, Alder, & Leese, 2004).

### Methodological considerations

Addressing some methodological considerations in the evaluation of our results is needed. The validity and transferability of this study was strengthened by the heterogeneity of our sample regarding age, duration of RA, and amount of daily leisure time SB. Furthermore, it was a methodological strength that the criterion of >4 h of daily leisure time SB had been self-reported by all patients before they were invited to the study. Accordingly, it was not the prospect of participating in an interview study that motivated them to state their daily leisure time SB. To enhance the validity further, efforts were made to promote feelings of confidence between patient and interviewer prior to the interviews. This was done by letting the patients choose locations for the interview and by a telephone conversation between interviewer and patient a couple of days before the interview. In addition, before starting the interview, the interviewer informed the patients that the purpose of the interview was not to judge them but was based on a genuine wish to let them freely describe their SB in order to gain knowledge on how patients with RA experience SB. Altogether, this may have made it more comfortable for the patients to narrate their experiences and increased the possibility of catching real experiences of SB during waking hours. Nevertheless, we cannot verify that the patients have described their waking SB exclusively without including time spent sleeping during the day. However, and in order to maintain the reflexivity of the study, we made constant efforts to ask clarifying questions to elicit further descriptions such as; “Please, tell me more” and “Please, give me an example.” Furthermore, the analytic process was followed rigorously and the findings were discussed concurrently between authors TT and BAE to ensure that they were grounded in the patients’ descriptions of reasons for SB rather than the authors’ preconceptions of their motives for SB. Regarding transferability, with reference to the general sex distribution in RA with two-thirds represented by women, the sex distribution is appropriate. Our study provides an understanding of how SB is experienced in patients with RA and provides important information to direct future intervention studies targeting day-to-day physical activity in patients with RA. Additionally, the results from this study may be transferred to other patients with a musculoskeletal disease experiencing pain, fatigue, and disability.

## Conclusions

Living with RA can be seen as a dynamic and balanced process including constant modification of daily physical activity level. Some days, pain and fatigue are barriers to performing valued activities and cause comprehensive sitting time. Although SB is not needed at times when RA is in remission, it still acts as a way of managing everyday life. In addition, personal and social factors lead to many hours spent sitting. The results from this study may provide guidance for clinicians on important issues that need to be addressed such as the new health promotion strategy of reducing SB, rather than to solely promote physical activity of moderate to hard intensity. Furthermore, based on results from this study, future research studies need to include intervention studies that should be individualized and tailored in order to encourage patients’ motivation towards a sustainable change in health behaviour.
